# Negative Modulation of the Metabotropic Glutamate Receptor Type 5 as a Potential Therapeutic Strategy in Obesity and Binge-Like Eating Behavior

**DOI:** 10.3389/fnins.2021.631311

**Published:** 2021-02-10

**Authors:** Tadeu P. D. Oliveira, Bruno D. C. Gonçalves, Bruna S. Oliveira, Antonio Carlos P. de Oliveira, Helton J. Reis, Claudia N. Ferreira, Daniele C. Aguiar, Aline S. de Miranda, Fabiola M. Ribeiro, Erica M. L. Vieira, András Palotás, Luciene B. Vieira

**Affiliations:** ^1^Departamento de Farmacologia, Instituto de Ciências Biológicas, Universidade Federal de Minas Gerais, Belo Horizonte, Brazil; ^2^Departamento de Morfologia, Instituto de Ciências Biológicas, Universidade Federal de Minas Gerais, Belo Horizonte, Brazil; ^3^Colégio Técnico, Universidade Federal de Minas Gerais, Belo Horizonte, Brazil; ^4^Departamento de Bioquimica e Imunologia, Instituto de Ciências Biológicas, Universidade Federal de Minas Gerais, Belo Horizonte, Brazil; ^5^Asklepios-Med (Private Medical Practice and Research Center), Szeged, Hungary; ^6^Institute of Fundamental Medicine and Biology, Kazan Federal University, Kazan, Russia

**Keywords:** obesity, mGluR5, high-fat, inflammation, glutamate

## Abstract

Obesity is a multifactorial disease, which in turn contributes to the onset of comorbidities, such as diabetes and atherosclerosis. Moreover, there are only few options available for treating obesity, and most current pharmacotherapy causes severe adverse effects, while offering minimal weight loss. Literature shows that metabotropic glutamate receptor 5 (mGluR5) modulates central reward pathways. Herein, we evaluated the effect of VU0409106, a negative allosteric modulator (NAM) of mGluR5 in regulating feeding and obesity parameters. Diet-induced obese C57BL/6 mice were treated for 14 days with VU0409106, and food intake, body weight, inflammatory/hormonal levels, and behavioral tests were performed. Our data suggest reduction of feeding, body weight, and adipose tissue inflammation in mice treated with high-fat diet (HFD) after chronic treatment with VU0409106. Furthermore, a negative modulation of mGluR5 also reduces binge-like eating, the most common type of eating disorder. Altogether, our results pointed out mGluR5 as a potential target for treating obesity, as well as related disorders.

## Graphical Abstract

Diet-induced obese (DIO) C57BL/6 mice were treated for 14 days with VU0409106, a negative allosteric modulator of mGluR5. Food intake, body weight, inflammatory/hormonal levels, and behavioral tests were performed. Our data suggest reduction of feeding, body weight, and adipose tissue inflammation in DIO mice after chronic treatment with VU0409106.

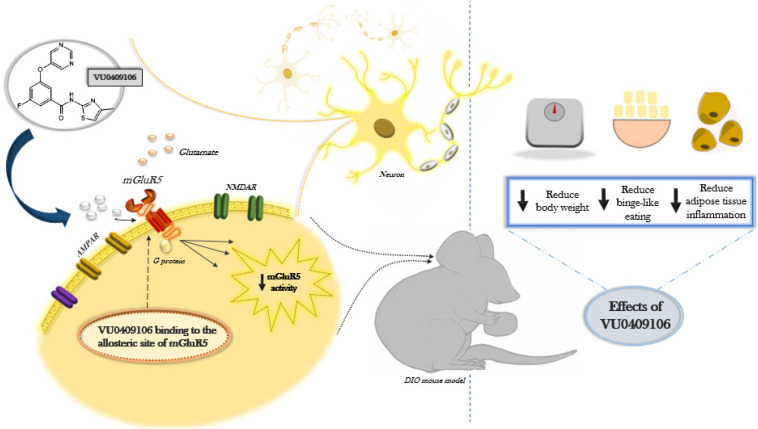

## Highlights

- VU0409106 is a negative allosteric modulator of mGluR5.
- VU0409106 reduces food consumption, body weight, and anxiety behavior of mice treated with high-fat diet.
- VU0409106 decreases inflammatory cytokines in the adipose tissue.
- VU0409106 treatment was able to reduce binge-like eating in mice.

## Introduction

Obesity prevalence is expanding in most countries according to the World Health Organization (Blüher, 2019). In fact, worldwide obesity numbers nearly tripled since 1975 (Blüher, 2019). Obesity is defined as an abnormal accumulation of body fat, associated with genetics, hormones, and environmental factors (González-Muniesa et al., 2017). The growing accessibility to highly palatable food, rich in fats and sugars, and reduced energy expenditure and sedentary lifestyle are considered the main environmental factors contributing to weight gain (Ravussin et al., 1988; Hill and Peters, 1998).

Obesity also increases the risk of several chronic conditions, including cardiovascular diseases, diabetes, cancer, psychiatric disorders, and many others (Kopelman, 2007). Changes leading to a series of physiological imbalances can be established in obese individuals, such as dyslipidemia and chronic inflammatory response (Dandona et al., 2004; Van Gaal et al., 2006). Besides, the progressive adipose tissue expansion in obesity is associated with insufficient angiogenesis, leading to hypoxia, cellular stress, oxidative damage, and necrosis, triggering an inflammatory response from adipocytes and local immune cells that becomes chronic in the context of long term obesity (Wood et al., 2009). Furthermore, a low-grade chronic inflammatory response can result in increased circulating levels of proinflammatory cytokines, leading to the onset of insulin resistance, diabetes, atherosclerosis, osteoarthritis, and so on (Dandona et al., 2004; Van Gaal et al., 2006; Kopelman, 2007; Wood et al., 2009).

Psychiatric disorders, including anxiety, depression, and binge eating, are also linked to obesity, yet there is still debate about whether they constitute cause or consequence of this disease (de Zwaan, 2001; Wurtman and Wurtman, 2018). Either way, efforts to reduce body weight and adiposity can be hampered by depression, anxiety, and other mood disorders, as these conditions can also promote weight gain (Collins et al., 2016). In agreement, some evidence shows a link between high-fat diet (HFD) consumption and the development of depression and anxiety, in animal models (Sharma and Fulton, 2013; Gancheva et al., 2017). Moreover, consumption of HFD might lead to neural adaptations in brain reward circuitry, a key area involved in binge eating and other addictive-like aspects of feeding behavior (Sharma and Fulton, 2013; Murray et al., 2014).

The metabotropic glutamate receptor 5 (mGluR5) is a G-protein–coupled receptor, associated with an intracellular rise in [Ca^2+^]_i_ through G_q_/G_11_ protein signaling, increasing the activity of PLC and the levels of IP_3_ and DAG (Niswender and Conn, 2010). There is evidence that mGluR5 may underlie obesity pathophysiology. For instance, the knockout of mGluR5 gene, as well as treatment with MTEP, a NAM of mGluR5, decreased body weight, plasma leptin and insulin levels, food intake, and feeding after food deprivation (Bradbury et al., 2005). Accordingly, CHPG, an mGluR5 agonist, stimulates food intake in mice, supporting a role of this receptor in mediating appetite and feeding (Ploj et al., 2010). Herein, we used a NAM of mGluR5, VU0409106, to investigate the effects of reducing mGluR5 activity over different aspects related to obesity, including binge-like eating. Compared to MTEP and related compounds, VU0409106 was recently synthesized, exhibiting improved pharmacokinetics and *in vivo* activity on reducing compulsion behavior without affecting motor behavior in mice (Varty et al., 2005; Koros et al., 2007; Felts et al., 2013).

Our results indicated that obese mice chronically injected with VU0409106 showed reduced body weight and adipose tissue inflammation. Locomotor activity and compulsive behavior were assessed, and the compound seems to reduce compulsive behavior in treated mice. Furthermore, our data confirm that the treatment with this drug was able to reduce binge-like eating in mice.

## Materials and Methods

### Animals

All procedures used in this study were approved and strictly followed the ethical principles of animal experimentation adopted by the Ethics Committee on Animal Use of Federal University of Minas Gerais and institutionally approved under protocol number 350/2015. Male C57BL/6 mice aging from 3 to 15 weeks were used. After weaning, mice were provided with *ad libitum* water and either HFD with 45% of total calories from fat, or control diet (CD) with 10% of total calories from fat (Supplementary Material). Diet was obtained from Rhoster^®^ Industry and Commerce (São Paulo, Brazil). All animals were housed in groups (two per box) and in conditions of 25°C, a 12 h light–dark cycle, with lights on 7 AM and off on 7 PM, and food and water *ad libitum*.

### Drugs

The compound VU0409106 was purchased from Tocris Bioscience^©^ (Bristol, United Kingdom). Fluoxetine hydrochloride was donated by Infinity Pharma^©^ (RJ, Brazil). The drugs were dissolved by sonication into vehicle (VEH) consisting of 10% Tween 80 (Sigma–Aldrich) and 90% physiological saline solution and administered via intraperitoneal route (IP). The doses of VU0409106 (50 mg) prepared were as follows: 3, 7.5, and 15 mg/kg. Fluoxetine, a selective serotonin reuptake inhibitor, was used at doses of 10 mg/kg as a positive control on reducing food consumption and body weight. After preparation, solutions were stored in the freezer at a temperature of −20°C until use.

### Diet-Induced Obesity

**FIGURE 1:**
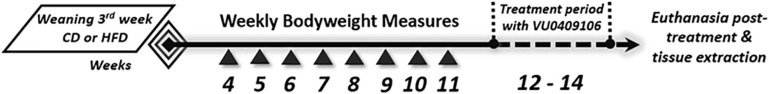
Scheme of diet-induced obesity (DIO) and chronic drug treatment. Three-week-old C57BL/6 mice were divided into control diet group (CD) and high-fat diet group (HFD). Animals were fed *ad libitum* throughout the experiment. At the 12th and 14th day, daily body weight and food intake were assessed for 14 days. In the last day of treatment, mice were submitted to behavioral experiments and afterward to biochemical analysis.

Mice were placed on HFD from weaning (third week), and body weight was measured weekly. At midweeks 12th and 14th, daily body weight and food intake were assessed in the morning (14 days). During this period, food intake was calculated by placing preweighed food in the cage daily. Consumption was normalized by mice body weight. At the beginning of the 15th week, mice were made to fast (7 AM–1 PM) and euthanized (Figure 1).

### Chronic Treatment in Obese Mice

The same protocol of diet-induced obesity (DIO) was used to assess the effects of 14 days treatment of obese mice with VU0409106 (Figure 1). Through the 12th to 14th week, mice were injected in the morning daily, via the IP route, with VEH, VU0409106 3 mg/kg, VU0409106 7.5 mg/kg, or fluoxetine 10 mg/kg. Those doses were based on previously reported data (Yen et al., 1987; Niswender and Conn, 2010; Felts et al., 2013). Daily food intake and body weight were measured, and after the period of treatment, the hypothalamus, serum, and epididymal adipose tissue were collected and properly stored for subsequent analysis.

### Fasting-Induced Food Intake Protocol

In order to assess the effects of VU0409106 on binge-like eating behavior, mice were fed with CD from weaning, and they were isolated in their cages at the beginning of the 11th week for 7 days for habituation. At the beginning of the 12th week, animals were deprived of food for 14 h (1 h prior to lights out, until 1 h after lights on). Thirty minutes before lights on, animals were treated with vehicle (VEH) or VU0409106 at doses of 3, 7.5, or 15 mg/kg or fluoxetine 10 mg/kg. After 14 h fasting, preweighed CD was reintroduced in cages, and food intake was measured at 15, 30, 60, and 210 min after refeeding. Food intake (milligrams) was normalized to body weight (grams).

### Intermittent HFD-Induced Food Intake Protocol

A second protocol to assess the effects of VU0409106 on binge-like eating behavior was conducted. Mice at 10 weeks of age were subjected to intermittent or continuous HFD, as described in the Supplementary Material. Previous work demonstrated that successive cycle of 24-h intermittent exposure to HFD induces binge-like eating in mice (Czyzyk et al., 2010). At the 12th week of age, these mice were injected with either VEH; VU0409106 at doses of 3, 7.5, or 15 mg/kg; or fluoxetine 10 mg/kg, 30 min prior to another presentation of preweighed HFD to the mice of the intermittent group. Food intake was measured 2.5 and 24 h after HFD presentation.

### Sample Processing

The hypothalamus and epididymal adipose tissue were rapidly collected and homogenized in an extraction solution (100 mg of tissue per milliliter), containing 0.4 M NaCl, 0.05% Tween 20, 0.5% bovine serum albumin, 0.1 mM phenyl methyl sulfonyl fluoride, 0.1 mM benzethonium chloride, 10 mM EDTA, and 20 KIU aprotinin, using Ultra-Turrax. Lysates were centrifuged at 13,000*g* for 10 min at 4°C; supernatants were collected and stocked at −70°C until use. Blood samples were also obtained, centrifuged at 1,500*g* for 10 min at 4°C, and the serum was collected and stocked at −70°C until use.

### Cytokines and Endocrine Markers Analysis

The concentration of the cytokines interleukin 6 (IL-6), IL-10, IL-12p70, interferon γ (IFN-γ), and tumor necrosis factor α (TNF-α), and chemokine monocyte chemoattractant protein 1 (MCP-1) was determined using a mouse CBA kit (BD Biosciences, San Diego, CA) and acquired on a FACS CANTO II flow cytometer (Becton Dickinson, San Jose, CA). The samples were incubated with capture microspheres (beads) covered by specifics antibodies to the respective cytokines and chemokines, as well as the proteins of the standard curve. Then, the color reagent was added, and the samples were incubated for 3 h, at room temperature, and protected from light. Then, the plate wells were washed with the Wash Buffer^®^ washing solution, provided in the kit, and subjected to centrifugation for 5 min at 200 rpm at room temperature. The supernatant was then aspirated and discarded. The precipitate containing the microspheres was then suspended with 300 μL of Wash Buffer^®^. The CBA results were analyzed by employing the software FCAP Array version 3.0 (Soft Flow Inc., Pécs, Hungary). Leptin, adiponectin, and insulin levels were detected by enzyme-linked immunosorbent assay (R&D Systems, Minneapolis, MN) in accordance to the manufacturer’s instructions. Total cholesterol and triglyceride assays were measured by specific kits from Bioclin^®^ (MG, Brazil). The monoreagent cholesterol and triglycerides kit are based on a colorimetric enzymatic test, in which a substrate is formed in which the color produced is directly proportional to the concentration of analyze, and its intensity is determined in a spectrophotometer at 500 nm.

### Glucose Measurement

All tests were performed in the morning with 6 h–fasted mice, and tail vein blood sample was collected. Blood glucose test was performed on mice at three different times: after weaning, before 14 days drug treatment, and after drug treatment. For measurements, *-chek active* device was used.

### Open-Field Test

To evaluate the locomotor activity, an open-field test was performed. In the afternoon, the animals were removed from the bioterium and taken to the test room, where the apparatus used in the experiment was located (the animals were not deprived from food or water). The habituation period was 30 min. After habituation, animals were placed in the apparatus (PhenoTyper^®^System; Noldus, Information Technology, Leesburg, VA, United States) and remained in the test for 30 min. The apparatus has an opaque plastic arena (30 × 30 cm), and animals could explore freely the entire area. The total distance traveled (cm) was analyzed using Ethovision XT software (Noldus, Information Technology, Leesburg, VA, United States).

### Marble-Burying Test

The basic protocol for this experiment was performed according to Nature Protocols (Deacon, 2006). For that, acrylic boxes measuring 29 × 17.5 cm in length, 20 spheres (marbles), and wood shavings were used. A quantity of wood shavings was placed, filling the boxes approximately 7 cm and forming a flat and compact surface. Twenty spheres were placed in rows (4 × 5) in the box on the shavings evenly spaced, with each space about 4 cm apart between the spheres. The animals faced the test in the morning. Before being taken to the experimental room, animals were kept for 30 min in packaging (there was no water or food deprivation). The drugs were administered 15 min before starting the behavioral test. The duration of the test was 30 min. The analyses were performed after the tests. The number of spheres buried and not buried was quantified. For the sphere to be considered buried, two-thirds of its dimension should be below the level of shavings. The same experimenter blindly analyzed all the tests performed.

### Statistics

Data are presented as mean ± SEM. All graphs and analyses were performed using GraphPad Prism 5.0 (GraphPad Software, San Diego). Before statistical tests, all data were analyzed by Grubbs ESD method for outlier detection, and extreme values were excluded from the analysis. Gaussian distribution of data was confirmed by Kolmogorov–Smirnov normality test. A comparison between two groups was performed by Student *t*-test. Three or more groups were analyzed by one-way analysis of variance (ANOVA) followed by Bonferroni *post hoc* test. The two-way ANOVA, followed by the Bonferroni *post hoc* was used in cases of two independent variables. In all cases, significance was defined by *p* < 0.05.

## Results

### DIO and Chronic Treatment With VU0409106 in Mice

Mice fed with HFD exhibited significantly higher body weight since the second week (two-way ANOVA, *F*_(2,23)_ = 10.52, *p* = 0.0004) (Supplementary Figure 1A). In addition, HFD group showed higher levels of total cholesterol (unpaired *t*-test, *t*_16_ = 2.335, *p* = 0.0329) (Supplementary Figure 1B), serum leptin (unpaired *t*-test, *t*_13_ = 2.184, *p* = 0.0479) (Supplementary Figure 1C), and also epididymal adipose tissue leptin levels (unpaired *t*-test, *t*_12_ = 5.584, *p* = 0.027) (Supplementary Figure 1D). Inflammatory markers in epididymal adipose tissue at the end of the DIO protocol were also measured, and higher levels of cytokines IL-12p70, TNF-α, IFN-γ, and the chemokine MCP-1 were detected (unpaired *t*-test, IL-12.70 *t*_10_ = 2.506, *p* = 0.0311; TNF-α *t*_10_ = 2.567, *p* = 0.028; IFN-γ *t*_10_ = 2.835, *p* = 0.0177; and MCP-1 *t*_11_ = 2.869, *p* = 0.0153) (Supplementary Figures 1E–J). Besides, results showed an increase in hypothalamic adiponectin levels in mice fed with HFD, although no other difference regarding serum insulin, adiponectin, triglycerides, and inflammatory markers outside epididymal adipose tissue was observed between HFD and control group (Table 1).

**TABLE 1:** Wild-type C57BL/6 mice after 11 weeks on HFD or CD.

	HFD	CD
Serum insulin (pg/mL)	1.725 ± 0.208	2.088 ± 0.239
Serum triglycerides (mg/dL)	61.38 ± 5.382	59.50 ± 8.379
Serum adiponectin (pg/mL)	188.9 ± 18.24	178.5 ± 27.57
Hypothalamic adiponectin (pg/mg)	25.68 ± 4.849*	12.42 ± 2.412
Adipose tissue adiponectin (pg/mg)	29.17 ± 7.737	36.91 ± 5.978
Hypothalamic IL-12p70 (pg/mg)	2.824 ± 0.686	2.822 ± 0.388
Hypothalamic TNF-α (pg/mg)	1.465 ± 0.170	1.551 ± 0.151
Hypothalamic IFN-γ (pg/mg)	0.481 ± 0.059	0.5415 ± 0.089
Hypothalamic MCP-1 (pg/mg)	8.536 ± 1.897	9.497 ± 1.517
Hypothalamic IL-10 (pg/mg)	1.510 ± 0.137	1.588 ± 0.154
Hypothalamic IL-6 (pg/mg)	1.364 ± 0.198	1.221 ± 0.116
Serum IL-12p70 (pg/mL)	17.27 ± 4.598	24.36 ± 6.843
Serum TNF-α (pg/mL)	15.33 ± 3.330	15.03 ± 3.186
Serum IFN-γ (pg/mL)	2.590 ± 0.332	3.106 ± 0.486
Serum MCP-1 (pg/mL)	94.64 ± 15.06	89.18 ± 15.02
Serum IL-10 (pg/mL)	15.35 ± 4.324	18.95 ± 4.613
Serum IL-6 (pg/mL)	7.391 ± 1.480	8.053 ± 1.475

*Statistically different from control diet–treated mice, p < 0.05, Student’s t-test, n = 6–8.

**FIGURE 2:**
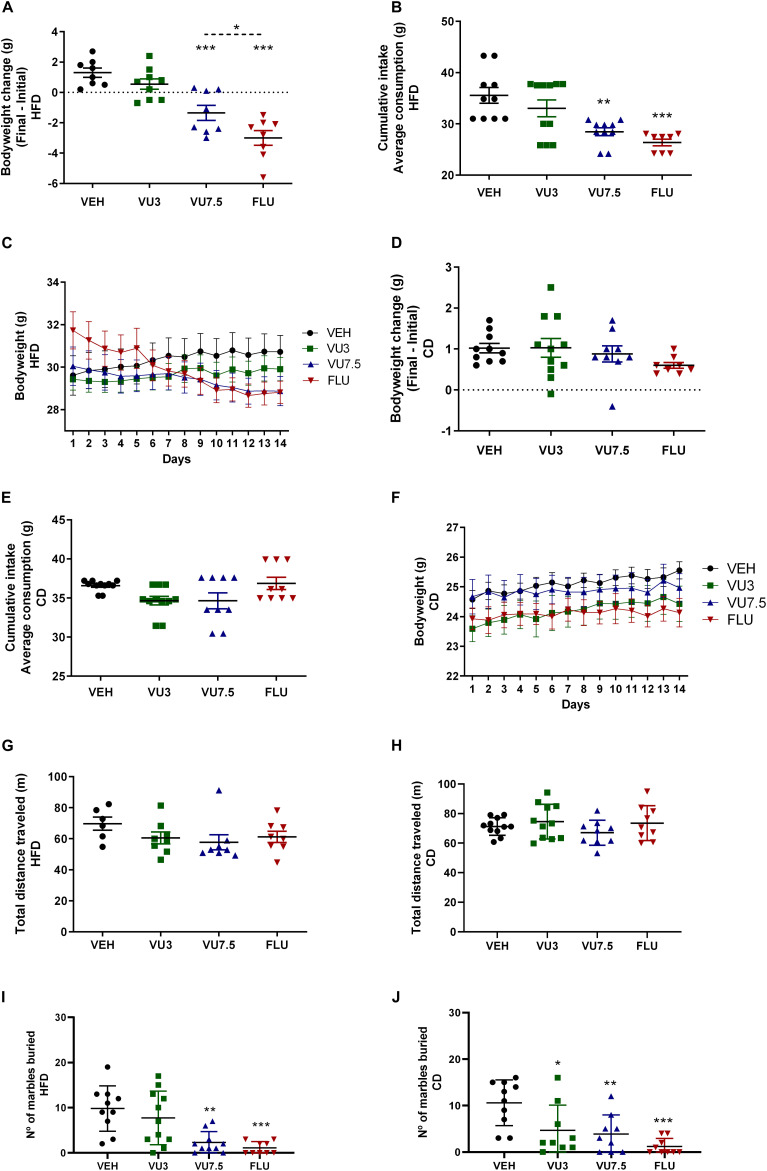
Effects of 14 days’ treatment on body weight, cumulative intake, and behavioral tests in HFD mice and CD mice. Scatter plot representation. **(A)** Treatment with VU0409106 7.5 mg/kg or fluoxetine 10 mg/kg reduced body weight of HFD mice (*n* = 8–9). **(B)** Food intake of HFD mice is decreased after treatment with VU0409106 7.5 mg/kg or fluoxetine 10 mg/kg (*n* = 8–11). **(C)** Graphical representation showing 14 days’ treatment on body weight of HFD mice. Treatment with VU0409106 (3 or 7.5 mg/kg) or fluoxetine (10 mg/kg) did not reduce body weight **(D)** or food intake **(E)** of CD mice (*n* = 8–11). **(F)** Graphical representation showing 14 days’ treatment on body weight of CD mice. **(G)** Treatment with VU0409106 7.5 mg/kg did not alter the distance traveled of HFD mice (*n* = 6–8). *p < 0.05, **p < 0.01, and ***p < 0.001 compared to vehicle after Bonferroni multiple-comparison *post hoc* test. **(H)** Treatment with VU0409106 (3 or 7.5 mg/kg) or fluoxetine (10 mg/kg) on CD mice did not change the distance traveled comparing to the control group. **(I)** VU0409106 7.5 mg/kg or fluoxetine 10 mg/kg reduced the number of marbles buried in HFD mice (*n* = 9–11). **p < 0.01 and ***p < 0.001 compared to vehicle after Bonferroni multiple-comparison *post hoc* test. **(J)** VU0409106 3 and 7.5 mg/kg or fluoxetine 10 mg/kg reduce the number of marbles buried in CD mice (*n* = 9–10). *p < 0.05, **p < 0.01, and ***p < 0.001 compared to vehicle after Bonferroni multiple-comparison *post hoc* test.

Interestingly, treatment with VU0409106 7.5 mg/kg or fluoxetine 10 mg/kg promoted weight loss (one-way ANOVA, *F*_(3,29)_ = 21.93, *p* < 0.001) and reduced food intake in HFD mice (one-way ANOVA, *F*_(3,35)_ = 9.654, *p* < 0.001) (Figures 2A–C). Besides, mice fed with CD were also treated with VU0409106, under the same protocol described for HFD mice. CD mice did not show significantly reduced body weight or food intake (Figures 2D–F). In order to investigate if drug treatment interferes with locomotor parameters of HFD and CD mice, the open-field test was performed. Importantly, HFD- or CD-treated mice with VU0409106 did not present reduction on total distance traveled compared to control group (Figures 2G,H). In addition to locomotor parameter, the effect of VU0409106 was evaluated on a model of compulsion (marble burying) in both groups. It was observed that HFD mice treated with VU0409106 7.5 mg/kg or fluoxetine 10 mg/kg buried fewer marbles compared to the control group (Figure 2I). Otherwise, CD mice treated with VU0409106 in all different concentrations or fluoxetine 10 mg/kg buried fewer marbles as compared to control group (Figure 2J).

**FIGURE 3:**
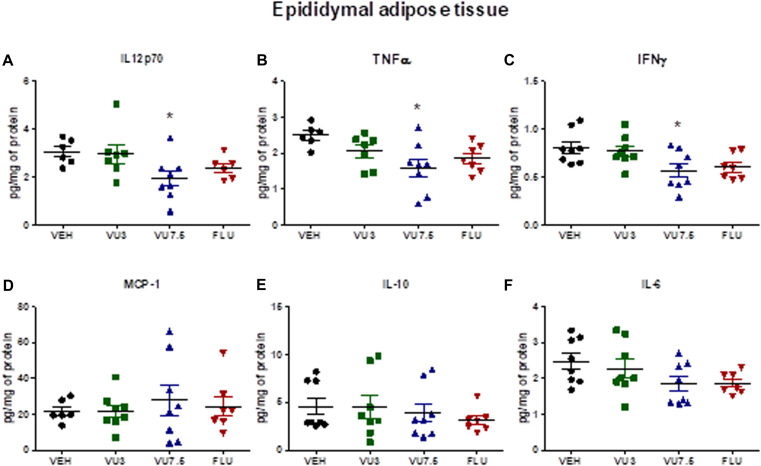
Decreased levels of inflammatory cytokines IL-12p70, TNF-α, and IFN-γ in the epididymal adipose tissue of HFD mice after treatment with VU0409106. Fourteen days’ treatment with VU0409106 7.5 mg/kg of HFD mice reduced **(A)** IL-12p70, **(B)** TNF-α, and **(C)** IFN-γ levels in the epididymal adipose tissue of HFD mice. **(D–F)** No effect of VU0409106 or fluoxetine on **(D)** MCP-1, **(E)** IL-10, or **(F)** IL-6 levels. The results are shown as mean ± *SD* from (*n* = 6–8). *p < 0.05 compared to vehicle after Bonferroni multiple-comparison *post hoc* test.

Nonetheless, we also observed a reduction of inflammatory cytokine levels IL-12p70, TNF-α, and IFN-γ in HFD mice treated with VU0409106 7.5 mg/kg in the adipose tissue of C57BL/6 obese mice (one-way ANOVA, IL-12p70 *F*_(3,23)_ = 3.116, *p* = 0.0459; TNF-α *F*_(3,24)_ = 3.904, *p* = 0.021; and IFN-γ *F*_(3,27)_ = 3.962, *p* = 0.0184) (Figures 3A–C). However, no difference was observed comparing vehicle-, VU0409106-, and fluoxetine-treated HFD mice in terms of leptin, adiponectin, total cholesterol, triglycerides, and inflammatory cytokine levels in hypothalamus or in serum (Supplementary Materials 2–6).

**FIGURE 4:**
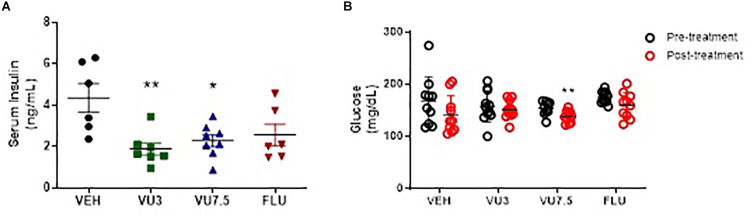
Fourteen days’ treatment with VU0409106 reduced serum insulin and glucose levels of HFD mice. **(A)** Scatter plot representation of treatment with VEH (vehicle), VU0409106 (3 or 7.5 mg/kg), and fluoxetine (10 mg/kg) (*n* = 7–8). *p < 0.05 and **p < 0.01 compared to vehicle after Bonferroni multiple-comparison *post hoc* test. **(B)** Glycaemia levels of HFD mice were measured prior (pretreatment) and after 14 days drug treatment (posttreatment). Blood samples were collected after 6-h fasting. VEH (vehicle), VU0409106 (3 or 7.5 mg/kg), and fluoxetine (10 mg/kg) (*n* = 10–11), **p < 0.01 compared to vehicle after Student’s *t*-test.

In addition, treatment with VU0409106 at both doses also reduced serum insulin levels in HFD mice (one-way ANOVA, *F*_(3,23)_ = 5.749, *p* = 0.0044) (Figure 4A), and VU0409106 7.5 mg/kg decreased glucose levels (unpaired *t*-test, *t*_17_ = 2.936, *p* = 0.0092) (Figure 4B).

### Effect of VU0409106 Treatment on Binge-Like Eating Behavior

**FIGURE 5:**
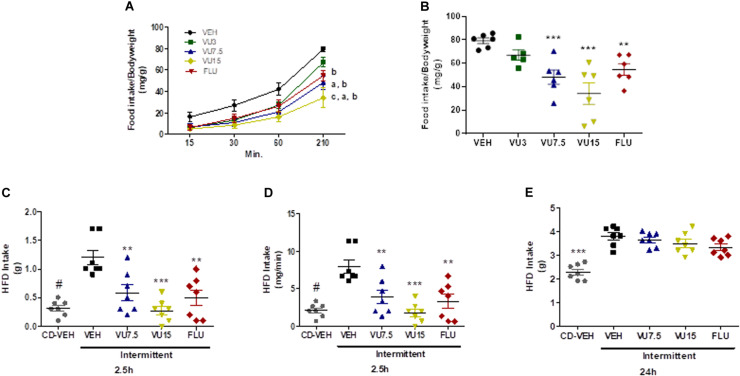
NAM VU0409106 reduced binge-like eating behavior in C57BL/6 mice. Fasting-induced food intake results are shown in A and B. **(A)** Food intake after 15, 30, 60, and 210 min of food return to the cage (a: different from vehicle at time 60; b: different from vehicle at time 210; and c: different from vehicle at time 30 after Bonferroni multiple-comparison *post hoc* test). **(B)** Food intake at 210 min (*n* = 6), **p < 0.01 and *** *p* < 0.001 compared to vehicle after Bonferroni multiple-comparison *post hoc* test. Intermittent-HFD-induced binge-like eating results are shown in **(C–E)**. HFD intakes were monitored **(C,D)** 2.5 h and **(E)** 24 h after presentation of HFD (*n* = 7), ^#^*p* < 0.05, **p < 0.01, and ***p < 0.001 after Bonferroni multiple-comparison *post hoc* test.

To assess the effects of negative mGluR5 modulation on fasting-induced food intake (Bradbury et al., 2005), 12 weeks-old non-obese mice were made to fast for 14 h (1 h prior to lights off until 1 h after lights on). Thirty minutes before returning food to the cages, mice were injected with vehicle, fluoxetine 10 mg/kg, or VU0409106 at doses of 3, 7.5, or 15 mg/kg. Food intake was measured 15, 30, 60, and 210 min after food was reintroduced and normalized by body weight. Mice injected with VU0409106 7.5 and 15 mg/kg showed reduced food intake after 30 min of returning food to the cage, as compared to vehicle-treated animals (Figure 5A) (two-way ANOVA; interaction *F*_(12,96)_ = 1.782, ns; group factor *F*_(3,96)_ = 16.74, *p* < 0.0001; time factor *F*_(4,96)_ = 105.8, *p* < 0.0001), and this reduction was even more pronounced at the end of the experiment (210 min after food presentation) (one-way ANOVA, *F*_(4,24)_ = 8.611, *p* = 0.0002) (Figure 5B). We also assessed binge-like eating behavior in a different protocol based on intermittent HFD exposure (Czyzyk et al., 2010). Ten-week-old non-obese mice were subjected to successive cycles of HFD and developed binge-like eating behavior (Supplementary Material). Thirty minutes before the third cycle of HFD exposure, mice were injected with vehicle, fluoxetine 10 mg/kg, or VU0409106 at 7.5 or 15 mg/kg. Food intake was measured 2.5 and 24 h later. As shown in Figure 5, mice with intermittent access to HFD had considerably higher food intake (one-way ANOVA, *F*_(4,30)_ = 17.99, *p* < 0.0001) (Figures 5C–E), including food intake per minute, which characterizes the binge-like eating behavior. At both tested doses of VU0409106, and also fluoxetine, results showed a reduction in binge-like eating 2.5 h after HFD presentation (Figures 5C,D). However, at 24 h, no difference was observed among drug-treated groups (Figure 5E).

## Discussion

In order to assess potential therapeutic applications of mGluR5-targeting drugs in obesity, herein we focused on the role of negative modulation of mGluR5 by VU0409106 in feeding regulation, body weight, binge eating, and adipose tissue inflammation on diet-induced obese mice. Importantly, VU0409106 is a drug structurally distinct from MTEP acting as a potent and selective NAM of mGluR5 binding at recognized allosteric binding site (Felts et al., 2013). This drug crosses the blood–brain barrier, displays good central nervous system (CNS) rates following intraperitoneal injection, and also reduces compulsive behavior in mice (Felts et al., 2013), which might be an interesting tool for treating CNS-related disorders, as binge eating in the context of obesity. Besides, rodents treated with VU0409106 differently from MTEP did not show altered locomotor activity, which may be related to alterations in learning and memory, social behavior deficits, and disruption on prepulse inhibition (Varty et al., 2005; Koros et al., 2007).

In the current study, we induced obesity in mice by using a protocol with 45% HFD. Our results showed that after HFD treatment, mice showed obesity features, such as increased body weight, cholesterol, leptin levels, and inflammatory markers. It is well known that adipose tissue inflammation is a hallmark of obesity and underlies several metabolic alterations associated with this condition (Dandona et al., 2004; Buettner et al., 2007; Wood et al., 2009). Accordingly, HFD mice showed elevated levels of the chemokine MCP-1 and inflammatory cytokines IL-12p70, TNF-α, and IFN-γ in the epididymal adipose tissue, 10 weeks after introduction of HFD. These findings reinforce the presence of an inflammatory milieu as described in obesity (Dandona et al., 2004; Huh et al., 2014). Moreover, the lack of higher levels of IL-10, a pivotal anti-inflammatory cytokine, points out the absence of a counter-regulatory process, which in turn may contribute to the chronic, low-grade inflammation typical of obesity (Juge-Aubry et al., 2005).

We also investigated the levels of adiponectin, a hormone produced by adipocytes, generally found at lower levels in obese subjects (Kern et al., 2003; Nigro et al., 2014). Although no difference was observed between HFD and CD mice regarding serum or adipose tissue levels of adiponectin, higher levels of this adipokine were found in the hypothalamus of obese mice. Based on the anti-inflammatory effects of adiponectin, its increased levels in the hypothalamus may explain, at least in part, the lack of a central inflammatory response in the HFD group (Huang et al., 2008; Nigro et al., 2014). In line with this finding, an intracerebroventricular injection of adiponectin was able to reverse the elevated proinflammatory signals in the hypothalamus of HFD mice, reinforcing adiponectin anti-inflammatory role in the brain in the obesity context (Koch et al., 2014).

It has been reported that the modulation of the glutamatergic system might be a promising strategy to treat obesity (Barja-Fernandez et al., 2014; Bojanowska and Ciosek, 2016). Accordingly, we investigated whether 14 days’ treatment with VU0409106 would oppose obesity in mice under HFD. The systemic administration of VU0409106 reversed weight gain and decreased food intake in HFD mice. Our data are in accordance with previous results showing that mGluR5 mediates appetite and energy balance in rodents (Bradbury et al., 2005). Also, previous data showed that MTEP, a NAM of mGluR5, decreased consumption of highly palatable food without altering consumption of the standard diet, which may explain, at least in part, the lack of treatment effect in CD mice (Bisaga et al., 2008). These results needed to be further investigated in order to clarify if the effect of VU0409016 is directly related to brain reward circuits. It is well known that eating behavior is directly affected by food taste and also that high-fat food is highly palatable (Rockwood and Bhathena, 1990; Melhorn et al., 2010; Small, 2012; Bake et al., 2014). Because of the difference in nutrient composition between diets, it is possible that VU0409106 acts by suppressing the intake of HFD more than CD as meal patterns are altered when mice are subjected to an HFD (Melhorn et al., 2010). Besides, mGluR5 is a diffuse receptor found in the brain, distributed in the following structures: olfactory bulb, anterior olfactory nucleus, olfactory tubercle, cerebral cortex, hippocampus, lateral septum, striatum, nucleus accumbens, inferior colliculus, and spinal trigeminal nucleus (Shigemoto et al., 1993). In addition, studies show that mGluR5 is expressed in several regions of the hypothalamus as in the preoptic, suprachiasmatic nucleus, and ventrolateral region (van den Pol et al., 1995). However, among these regions, mGluR5 is strongly expressed in the ventrolateral pole of the ventromedial nucleus (an area related to metabolic balance and food intake) (van den Pol et al., 1995). As mGluR5 are expressed in key brain areas controlling feeding behavior and rewarding brain circuits, as hypothalamus, nucleus accumbens, and dorsolateral striatum, it is possible that the negative modulation of these receptors reduced the rewarding aspects induced by the palatable food in the HFD group (van den Pol et al., 1995; Boer et al., 2010; Terbeck et al., 2015). In fact, there is evidence that glutamatergic antagonists seem to be more selective in decreasing the reinforcing effect of food, which is more related to hedonic than homeostatic processes (Saper et al., 2002). Moreover, it is quite tempting to postulate that the mechanisms underlying the effects of negative modulation of mGluR5 on reducing food intake may resemble the antagonist of those receptors on decreasing self-administration and drug-seeking behavior of agents, such as alcohol, cocaine, nicotine, and opiates (Paterson and Markou, 2005; Bäckström and Hyytiä, 2006; Osborne and Olive, 2008; Rutten et al., 2011; Wang et al., 2013).

In the behavioral tests performed, no uncommon results were found. As previously described, MPEP and fluoxetine decrease the compulsive behavior of burying marbles on mice (Spooren et al., 2000; Nicolas et al., 2006; Arora et al., 2013), and the compound VU0409106 itself has a similar ability (Felts et al., 2013). In the open-field test, according to our results VU0409106 administration did not cause hyperlocomotion or sedation in treated mice. However, more side effects of the compound need to be explored.

**FIGURE 6:**
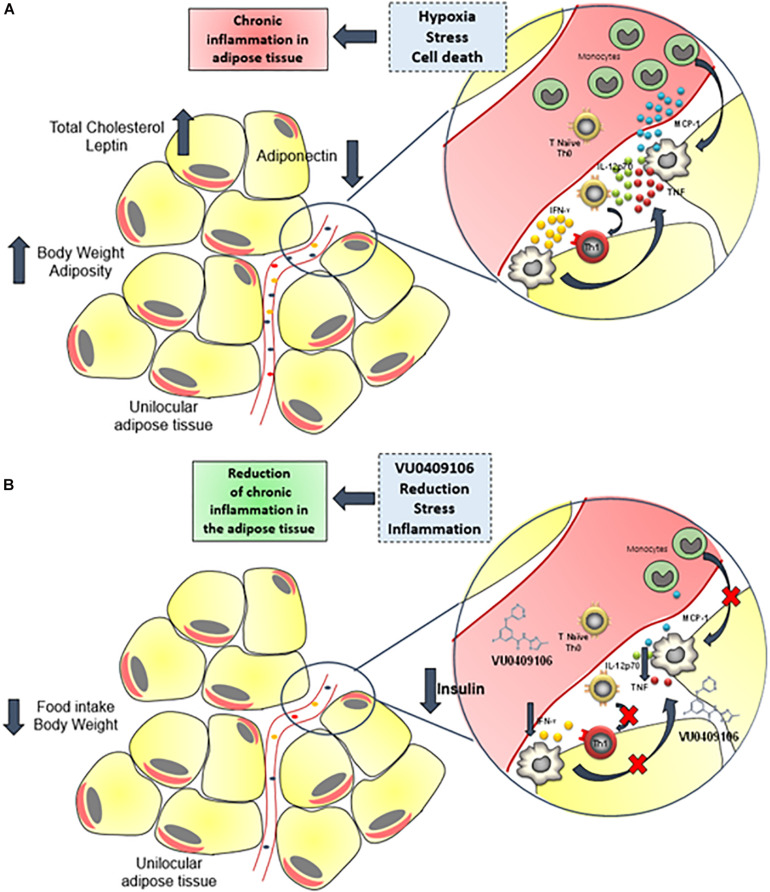
Anti-inflammatory role of VU0409106 in the adipose tissue of obese mice. **(A)** Increased adiposity may contribute to hypoxia, stress, and cell death, which triggers an inflammatory response with enhanced local levels of inflammatory mediators, especially cytokines such as IL-12p70, TNF-α, and IFN-γ and the chemokine MCP-1 and consequent immune cell recruitment. As a result, a chronic, low-grade inflammation of adipose tissue is sustained. **(B)** Chronic administration of VU04090106 reduces food consumption and body weight and promotes an anti-inflammatory milieu.

The negative modulation of mGluR5 by VU04090106 also decreased the adipose tissue inflammation induced by HFD. It is unclear how VU0409106 decreased inflammatory markers selectively in the adipose tissue without affecting their levels in the serum or hypothalamus. Nonetheless, VU0490106-associated decrease in inflammatory signaling in the epididymal adipose tissue of HFD mice may also be dependent on the body weight loss alongside with reduced adiposity due to decreased food intake, which in turn leads to diminished stress upon adipocytes and to a decrease in local inflammation (Figure 6). Corroborating our findings, anti-inflammatory effects, including decrease in the expression of inflammatory cytokines, as well as in the recruitment of immune cells to the lesion site, have been also reported following negative regulation of mGluR5 in other pathological conditions, such as traumatic brain injury (Byrnes et al., 2009; Yang et al., 2017). Considering the widespread distribution of mGluR5 (e.g., CNS, liver, hepatocytes, thymus, immune cells), further studies are necessary to address the mechanisms behind VU0490106 anti-inflammatory properties in obesity (Boldyrev et al., 2005; Ferrigno et al., 2017).

An intriguing effect was observed in the serum insulin and glucose levels of HFD mice in response to the treatment with VU0409106 (Figure 6B). Although no difference in serum insulin level was observed between HFD and CD mice, only HFD mice had lower insulin and glucose levels after VU0409106 treatment. Likewise, lower serum insulin level after mGluR5 negative modulation was observed only in HFD mice as reported by Bradbury et al. (2005). Evidence suggests that mGluR is involved in the regulation of hormone secretion in the endocrine pancreas (Brice et al., 2002; Storto et al., 2006). The mGluR5 appears also to be required for an optimal insulin response to glucose both in clonal beta cells and mice (Storto et al., 2006). In clonal pancreatic beta cells, mGluR5 is expressed at the cell surface and also found in purified insulin-containing granules (Storto et al., 2006). Moreover, the NAM of mGluR5, MPEP, was able to inhibit glucose-stimulated [Ca^2+^]_i_ increase and insulin secretion in those cells (Storto et al., 2006) supporting our findings.

Binge-eating disorder is strongly linked with obesity, and binge eating *per se* is associated with a high burden of metabolic risk factors in the general population (Succurro et al., 2015; Davis, 2017). Binge-eating disorder has genetic and environmental components, being also associated with abnormalities in main cognitive areas as prefrontal cortex and the striatum (Kessler et al., 2016). In addition, glutamatergic neurotransmission is an interesting target for binge-eating disorder treatment, as it plays a role in reinforcing action of natural stimuli, such as food and drug abuse (Bisaga et al., 2008; Gass and Olive, 2008; Kalivas, 2009; Guardia et al., 2011). Thus, in order to assess the effects of negative modulation of mGluR5 in a model of binge-eating disorder, we selected two different protocols: a fasting protocol and an intermittent HFD access model, which does not involve the stress of forced fasting (Smith and Robbins, 2013). There is evidence that different neural substrates may control hunger- and non–hunger-driven food intake (Cao et al., 2014; Xu et al., 2017). Our results showed that in both protocols, the NAM VU0409106 was able to reduce the binge-like eating behavior. Fluoxetine, a selective inhibitor of reuptake of serotonin, which is largely used in the treatment of binge-eating disorders, was also effective in reducing binge-like eating in our models (Arnold et al., 2002; Guardia et al., 2011; Li et al., 2015). In addition, the role of mGluR5 on eating disorders was assessed by several authors (Bradbury et al., 2005; Bisaga et al., 2008; Guardia et al., 2011). More recently, interesting work described the part of mGluR5, *in vivo*, in bulimia nervosa (Mihov et al., 2020). In this study, the authors found higher distribution volume of mGluR5 in areas linked to processing emotional and cognitive information related to self-control, as anterior cingulate cortex and medial orbitofrontal cortex in individuals with bulimia (Mihov et al., 2020). Overall, our data confirm the role of mGluR5 on eating disorders and paved the way for the development of therapeutic strategies focused in the glutamatergic transmission for the treatment of this condition.

It is also important to highlight that our DIO model used small laboratory animals, which have different feeding patterns and compensatory mechanisms than humans, which may limit the extrapolation of the findings. And besides the limitations of this study on lacking on a deeper investigation of the mechanisms behind VU0409106 weight loss and decreased inflammatory markers on the adipose tissue, our findings may have important clinical implications. The current study confirms the role of mGluR5 on feeding and body weight regulation, pointing out this glutamatergic receptor as an important clinical target to treat obesity and related disorders. Further research and prospective studies, however, are needed to assess and evaluate the potential of VU0409106 on long-term treatment.

## Conclusion

This work provides first evidence of positive results on the mGluR5 NAM, VU0409106, in DIO and binge-like eating models. The weight loss achieved by VU0409106 administration was restricted to obese mice; likewise, the reduced food intake. The compound also reduced epididymal adipose tissue inflammation, suggesting that it could hold other therapeutic effects in the context of obesity and metabolic syndromes. We also showed that both hunger- and non–hunger-driven food intake, resembling binge-like eating, are suppressed by VU0409106-negative mGluR5 modulation. In summary, this work adds to previous evidence linking potential therapeutic application of mGluR5 antagonists to obesity and obesity-related disorders, paving the way for the development of translational approaches and promised treatments.

## Data Availability Statement

The original data presented in the study are included in the article/Supplementary Material, further inquiries can be directed to the corresponding author.

## Ethics Statement

The studies involving animals were reviewed and approved by Ethics Committee on Animal Use of Federal University of Minas Gerais and institutionally approved under protocol number 350/2015.

## Author Contributions

LV designed the study. BG and TO performed most diet and behavioral experiments. BO, AM, and EV performed Elisa and CBA assays. CF performed analysis of triglycerides and cholesterol. DA contributed to the conception and design of diet protocols of the study. FR, HR, AO, and AP made substantial contributions to conception and design of the study and critically revised the manuscript for important intellectual content. BG, TO, and LV analyzed the results and wrote the article. All other authors revised the data and discussed the manuscript.

## Conflict of Interest

The authors declare that the research was conducted in the absence of any commercial or financial relationships that could be construed as a potential conflict of interest.

## Abbreviations

HFD: High-fat diet
CHPG: 2-Chloro-5-hydroxyphenylglycine
CD: Control diet
CNS: Central nervous system
mGluR5: Metabotropic glutamate receptor 5
PLC: Phospholipase C
IP3: Inositol 3-phosphate
DAG: Diacylglycerol
NAM: Negative allosteric modulator
DIO: Diet-induced obesity/diet-induced obese
TNF: Tumor necrosis factor
IFN- γ: Interferon gamma
IL: Interleukin
MCP-1: Monocyte chemoattractant protein
VEH: Vehicle
IP: Intraperitoneal route
MTEP: [3-[(2-Methyl-1,3-thiazol-4-yl)-ethynyl]-pyridine.

## References

[B1] Arnold L. M. McElroy S. L. Hudson J. I. Welge J. A. Bennett A. J. Keck P. E. (2002). A placebo-controlled, randomized trial of fluoxetine in the treatment of binge-eating disorder. *J. Clin. Psychiatry* 63 1028–1033. 10.4088/jcp.v63n1113 12444817

[B2] Arora T. Bhowmik M. Khanam R. Vohora D. (2013). Oxcarbazepine and fluoxetine protect against mouse models of obsessive compulsive disorder through modulation of cortical serotonin and CREB pathway. *Behav. Brain Res.* 247 146–152. 10.1016/j.bbr.2013.02.038 23473877

[B3] Bäckström P. Hyytiä P. (2006). Ionotropic and metabotropic glutamate receptor antagonism attenuates cue-induced cocaine seeking. *Neuropsychopharmacology* 31 778–786. 10.1038/sj.npp.1300845 16123768

[B4] Bake T. Murphy M. Morgan D. G. Mercer J. G. (2014). Large, binge-type meals of high fat diet change feeding behaviour and entrain food anticipatory activity in mice. *Appetite* 77 60–71. 24631639 10.1016/j.appet.2014.02.020 PMC4152876

[B5] Barja-Fernandez S. Leis R. Casanueva F. F. Seoane L. M. (2014). Drug development strategies for the treatment of obesity: how to ensure efficacy, safety, and sustainable weight loss. *Drug Des. Devel. Ther.* 8 2391–2400. 10.2147/dddt.s53129 25489237 PMC4257050

[B6] Bisaga A. Danysz W. Foltin R. W. (2008). Antagonism of glutamatergic NMDA and mGluR5 receptors decreases consumption of food in baboon model of binge-eating disorder. *Eur. Neuropsychopharmacol.* 18 794–802. 10.1016/j.euroneuro.2008.05.004 18573641 PMC2591926

[B7] Blüher M. (2019). Obesity: global epidemiology and pathogenesis. *Nat. Rev. Endocrinol.* 15 288–298. 10.1038/s41574-019-0176-8 30814686

[B8] Boer K. Encha-Razavi F. Sinico M. Aronica E. (2010). Differential distribution of group I metabotropic glutamate receptors in developing human cortex. *Brain Res.* 1324 24–33. 10.1016/j.brainres.2010.02.005 20149785

[B9] Bojanowska E. Ciosek J. (2016). Can we selectively reduce appetite for energy-dense foods? an overview of pharmacological strategies for modification of food preference behavior. *Curr. Neuropharmacol.* 14 118–142. 10.2174/1570159x14666151109103147 26549651 PMC4825944

[B10] Boldyrev A. A. Carpenter D. O. Johnson P. (2005). Emerging evidence for a similar role of glutamate receptors in the nervous and immune systems. *J. Neurochem.* 95 913–918. 10.1111/j.1471-4159.2005.03456.x 16271044

[B11] Bradbury M. J. Campbell U. Giracello D. Chapman D. King C. Tehrani L. (2005). Metabotropic glutamate receptor mGlu5 is a mediator of appetite and energy balance in rats and mice. *J. Pharmacol. Exp. Ther.* 313 395–402. 10.1124/jpet.104.076406 15590770

[B12] Brice N. L. Varadi A. Ashcroft S. J. Molnar E. (2002). Metabotropic glutamate and GABA(B) receptors contribute to the modulation of glucose-stimulated insulin secretion in pancreatic beta cells. *Diabetologia* 45 242–252. 10.1007/s00125-001-0750-0 11935156

[B13] Buettner R. Scholmerich J. Bollheimer L. C. (2007). High-fat diets: modeling the metabolic disorders of human obesity in rodents. *Obesity (Silver Spring)* 15 798–808. 10.1038/oby.2007.608 17426312

[B14] Byrnes K. R. Stoica B. Loane D. J. Riccio A. Davis M. I. Faden A. I. (2009). Metabotropic glutamate receptor 5 activation inhibits microglial associated inflammation and neurotoxicity. *Glia* 57 550–560. 10.1002/glia.20783 18816644 PMC2644739

[B15] Cao X. Xu P. Oyola M. G. Xia Y. Yan X. Saito K. (2014). Estrogens stimulate serotonin neurons to inhibit binge-like eating in mice. *J. Clin. Invest.* 124 4351–4362. 10.1172/jci74726 25157819 PMC4191033

[B16] Collins J. Meng C. Eng A. (2016). Psychological impact of severe obesity. *Curr. Obes. Rep.* 5 435–440. 10.1007/s13679-016-0229-4 27679429

[B17] Czyzyk T. A. Sahr A. E. Statnick M. A. (2010). A model of binge-like eating behavior in mice that does not require food deprivation or stress. *Obesity (Silver Spring)* 18 1710–1717. 10.1038/oby.2010.46 20300082

[B18] Dandona P. Aljada A. Bandyopadhyay A. (2004). Inflammation: the link between insulin resistance, obesity and diabetes. *Trends Immunol.* 25 4–7. 10.1016/j.it.2003.10.013 14698276

[B19] Davis C. (2017). A commentary on the associations among ‘food addiction’, binge eating disorder, and obesity: overlapping conditions with idiosyncratic clinical features. *Appetite* 115 3–8. 10.1016/j.appet.2016.11.001 27816464

[B20] de Zwaan M. (2001). Binge eating disorder and obesity. *Int. J. Obes. Relat. Metab. Disord.* 25(Suppl. 1) S51–S55. 11466589 10.1038/sj.ijo.0801699

[B21] Deacon R. M. (2006). Digging and marble burying in mice: simple methods for in vivo identification of biological impacts. *Nat. Protoc.* 1 122–124. 10.1038/nprot.2006.20 17406223

[B22] Felts A. S. Rodriguez A. L. Morrison R. D. Venable D. F. Manka J. T. Bates B. S. (2013). Discovery of VU0409106: a negative allosteric modulator of mGlu5 with activity in a mouse model of anxiety. *Bioorg. Med. Chem. Lett.* 23 5779–5785. 10.1016/j.bmcl.2013.09.001 24074843 PMC3846293

[B23] Ferrigno A. Berardo C. Di Pasqua L. G. Siciliano V. Richelmi P. Vairetti M. (2017). Localization and role of metabotropic glutamate receptors subtype 5 in the gastrointestinal tract. *World J. Gastroenterol.* 23 4500–4507. 10.3748/wjg.v23.i25.4500 28740338 PMC5504365

[B24] Gancheva S. Galunska B. Zhelyazkova-Savova M. (2017). Diets rich in saturated fat and fructose induce anxiety and depression-like behaviours in the rat: is there a role for lipid peroxidation? *Int. J. Exp. Pathol.* 98 296–306. 10.1111/iep.12254 29210119 PMC5743787

[B25] Gass J. T. Olive M. F. (2008). Glutamatergic substrates of drug addiction and alcoholism. *Biochem. Pharmacol.* 75 218–265. 10.1016/j.bcp.2007.06.039 17706608 PMC2239014

[B26] González-Muniesa P. Mártinez-González M. Hu F. Després J. P. Matsuzawa Y. Loos R. J. F. (2017). Obesity. *Nat. Rev. Dis. Prim.* 3 1–18. 10.1038/nrdp.2017.34 28617414

[B27] Guardia D. Rolland B. Karila L. Cottencin O. (2011). GABAergic and glutamatergic modulation in binge eating: therapeutic approach. *Curr. Pharm. Des.* 17 1396–1409. 10.2174/138161211796150828 21524265

[B28] Hill J. O. Peters J. C. (1998). Environmental contributions to the obesity epidemic. *Science* 280 1371–1374. 10.1126/science.280.5368.1371 9603719

[B29] Huang H. Park P. H. McMullen M. R. Nagy L. E. (2008). Mechanisms for the anti-inflammatory effects of adiponectin in macrophages. *J Gastroenterol. Hepatol.* 23(Suppl. 1) S50–S53. 18336664 10.1111/j.1440-1746.2007.05284.x

[B30] Huh J. Y. Park Y. J. Ham M. Kim J. B. (2014). Crosstalk between adipocytes and immune cells in adipose tissue inflammation and metabolic dysregulation in obesity. *Mol. Cells* 37 365–371. 10.14348/molcells.2014.0074 24781408 PMC4044307

[B31] Juge-Aubry C. E. Somm E. Pernin A. Alizadeh N. Giusti V. Dayer J. M. (2005). Adipose tissue is a regulated source of interleukin-10. *Cytokine* 29 270–274. 15749027 10.1016/j.cyto.2004.10.017

[B32] Kalivas P. W. (2009). The glutamate homeostasis hypothesis of addiction. *Nat. Rev. Neurosci.* 10 561–572. 10.1038/nrn2515 19571793

[B33] Kern P. A. di Gregorio G. B. Lu T. Rassouli N. Ranganathan G. (2003). Adiponectin expression from human adipose tissue: relation to obesity, insulin resistance, and tumor necrosis factor-α expression. *Diabetes* 52 1779–1785. 10.2337/diabetes.52.7.1779 12829646

[B34] Kessler R. M. Hutson P. H. Herman B. K. Potenza M. N. (2016). The neurobiological basis of binge-eating disorder. *Neurosci. Biobehav. Rev.* 63 223–238. 10.1016/j.neubiorev.2016.01.013 26850211

[B35] Koch C. E. Lowe C. Legler K. Benzler J. Boucsein A. Böttiger G. (2014). Central adiponectin acutely improves glucose tolerance in male mice. *Endocrinology* 155 1806–1816. 10.1210/en.2013-1734 24564394

[B36] Kopelman P. (2007). Health risks associated with overweight and obesity. *Obes. Rev.* 8(Suppl. 1) 13–17. 10.1111/j.1467-789x.2007.00311.x 17316295

[B37] Koros E. Rosenbrock H. Birk G. Weiss C. Sams-Dodd F. (2007). The selective mGlu5 receptor antagonist MTEP, similar to NMDA receptor antagonists, induces social isolation in rats. *Neuropsychopharmacology* 32 562–576. 10.1038/sj.npp.1301133 16794564

[B38] Li B. Shao D. Luo Y. Wang P. Liu C. Zhang X. (2015). Role of 5-HT3 receptor on food intake in fed and fasted mice. *PLoS One* 10:e0121473. 10.1371/journal.pone.0121473 25789930 PMC4366218

[B39] Melhorn S. J. Krause E. G. Scott K. A. Mooney M. R. Johnson J. D. Woods S. C. (2010). Acute exposure to a high-fat diet alters meal patterns and body composition. *Physiol. Behav.* 99 33–39. 10.1016/j.physbeh.2009.10.004 19835896 PMC2794977

[B40] Mihov Y. Treyer V. Akkus F. Toman E. Milos G. Ametamey S. M. (2020). Metabotropic glutamate receptor 5 in bulimia nervosa. *Sci. Rep.* 10:6374. 10.1038/s41598-020-63389-7 PMC7156702 32286451

[B41] Murray S. Tulloch A. Gold M. S. Avena N. M. (2014). Hormonal and neural mechanisms of food reward, eating behaviour and obesity. *Nat. Rev. Endocrinol.* 10 540–552. 10.1038/nrendo.2014.91 24958311

[B42] Nicolas L. B. Kolb Y. Prinssen E. P. (2006). A combined marble burying-locomotor activity test in mice: a practical screening test with sensitivity to different classes of anxiolytics and antidepressants. *Eur. J. Pharmacol.* 547 106–115. 10.1016/j.ejphar.2006.07.015 16934246

[B43] Nigro E. Scudiero O. Monaco M. L. Palmieri A. Mazzarella G. Costagliola C. (2014). New insight into adiponectin role in obesity and obesity-related diseases. *Biomed. Res. Int.* 2014:658913. 10.1155/2014/658913 PMC4109424 25110685

[B44] Niswender C. M. Conn P. J. (2010). Metabotropic glutamate receptors: physiology, pharmacology, and disease. *Annu. Rev. Pharmacol. Toxicol.* 50 295–322. 10.1146/annurev.pharmtox.011008.145533 20055706 PMC2904507

[B45] Osborne M. P. Olive M. F. (2008). A role for mGluR5 receptors in intravenous methamphetamine self-administration. *Ann. N. Y. Acad. Sci.* 1139 206–211. 10.1196/annals.1432.034 18991866

[B46] Paterson N. E. Markou A. (2005). The metabotropic glutamate receptor 5 antagonist MPEP decreased break points for nicotine, cocaine and food in rats. *Psychopharmacology (Berl)* 179 255–261. 10.1007/s00213-004-2070-9 15619120

[B47] Ploj K. Albery-Larsdotter S. Arlbrandt S. Kjaer M. B. Skantze P. M. Storlien L. H. (2010). The metabotropic glutamate mGluR5 receptor agonist CHPG stimulates food intake. *Neuroreport* 21 704–708. 20505551 10.1097/WNR.0b013e32833b4fe7

[B48] Ravussin E. Lillioja S. Knowler W. C. Christin L. Freymond D. Abbott W. G. (1988). Reduced rate of energy expenditure as a risk factor for body-weight gain. *N. Engl. J. Med.* 318 467–472. 10.1056/nejm198802253180802 3340128

[B49] Rockwood G. A. Bhathena S. J. (1990). High-fat diet preference in developing and adult rats. *Physiol. Behav.* 48 79–82. 10.1016/0031-9384(90)90264-5 2236281

[B50] Rutten K. Van Der Kam E. L. De Vry J. Bruckmann W. Tzschentke T. M. (2011). The mGluR5 antagonist 2-methyl-6-(phenylethynyl)-pyridine (MPEP) potentiates conditioned place preference induced by various addictive and non-addictive drugs in rats. *Addict. Biol.* 16 108–115. 10.1111/j.1369-1600.2010.00235.x 20579001

[B51] Saper C. B. Chou T. C. Elmquist J. K. (2002). The need to feed: homeostatic and hedonic control of eating. *Neuron* 36 199–211. 12383777 10.1016/s0896-6273(02)00969-8

[B52] Sharma S. Fulton S. (2013). Diet-induced obesity promotes depressive-like behaviour that is associated with neural adaptations in brain reward circuitry. *Int. J. Obes. (Lond)* 37 382–389. 10.1038/ijo.2012.48 22508336

[B53] Shigemoto R. Nomura S. Ohishi H. Sugihara H. Nakanishi S. Mizuno N. (1993). Immunohistochemical localization of a metabotropic glutamate receptor, mGluR5, in the rat brain. *Neurosci. Lett*. 163 53–57. 10.1016/0304-3940(93)90227-c 8295733

[B54] Small D. M. (2012). Flavor is in the brain. *Physiol Behav.* 107 540–552. 22542991 10.1016/j.physbeh.2012.04.011

[B55] Smith D. G. Robbins T. W. (2013). The neurobiological underpinnings of obesity and binge eating: a rationale for adopting the food addiction model. *Biol. Psychiatry* 73 804–810. 10.1016/j.biopsych.2012.08.026 23098895

[B56] Spooren W. P. Vassout A. Neijt H. C. Kuhn R. Gasparini F. Roux S. (2000). Anxiolytic-like effects of the prototypical metabotropic glutamate receptor 5 antagonist 2-methyl-6-(phenylethynyl)pyridine in rodents. *J. Pharmacol. Exp. Ther.* 295 1267–1275. 11082464

[B57] Storto M. Capobianco L. Battaglia G. Molinaro G. Gradini R. Riozzi B. (2006). Insulin secretion is controlled by mGlu5 metabotropic glutamate receptors. *Mol. Pharmacol.* 69 1234–1241. 10.1124/mol.105.018390 16424079

[B58] Succurro E. Segura-Garcia C. Ruffo M. Caroleo M. Rania M. Aloi M. (2015). Obese patients with a binge eating disorder have an unfavorable metabolic and inflammatory profile. *Medicine (Baltimore)* 94:e2098. 10.1097/md.0000000000002098 26717356 PMC5291597

[B59] Terbeck S. Akkus F. Chesterman L. P. Hasler G. (2015). The role of metabotropic glutamate receptor 5 in the pathogenesis of mood disorders and addiction: combining preclinical evidence with human positron emission tomography (PET) studies. *Front. Neurosci.* 9:86. 10.3389/fnins.2015.00086 25852460 PMC4364244

[B60] van den Pol A. N. Romano C. Ghosh P. (1995). Metabotropic glutamate receptor mGluR5 subcellular distribution and developmental expression in hypothalamus. *J. Comp. Neurol.* 362 134–150. 10.1002/cne.903620108 8576426

[B61] Van Gaal L. F. Mertens I. L. De Block C. E. (2006). Mechanisms linking obesity with cardiovascular disease. *Nature* 444 875–880. 10.1038/nature05487 17167476

[B62] Varty G. B. Grilli M. Forlani A. Fredduzzi S. Grzelak M. E. Guthrie D. H. (2005). The antinociceptive and anxiolytic-like effects of the metabotropic glutamate receptor 5 (mGluR5) antagonists, MPEP and MTEP, and the mGluR1 antagonist, LY456236, in rodents: a comparison of efficacy and side-effect profiles. *Psychopharmacology (Berl)* 179 207–217. 10.1007/s00213-005-2143-4 15682298

[B63] Wang X. Moussawi K. Knackstedt L. Shen H. Kalivas P. W. (2013). Role of mGluR5 neurotransmission in reinstated cocaine-seeking. *Addict. Biol.* 18 40–49. 10.1111/j.1369-1600.2011.00432.x 22340009 PMC3360124

[B64] Wood I. S. de Heredia F. P. Wang B. Trayhurn P. (2009). Cellular hypoxia and adipose tissue dysfunction in obesity. *Proc. Nutr. Soc.* 68 370–377. 10.1017/s0029665109990206 19698203

[B65] Wurtman J. Wurtman R. (2018). The trajectory from mood to obesity. *Curr. Obes. Rep.* 7 1–5. 10.1007/s13679-017-0291-6 29218451 PMC5829131

[B66] Xu P. He Y. Cao X. Valencia-Torres L. Yan X. Saito K. (2017). Activation of serotonin 2C receptors in dopamine neurons inhibits binge-like eating in mice. *Biol. Psychiatry.* 81 737–747. 10.1016/j.biopsych.2016.06.005 27516377 PMC5148733

[B67] Yang T. Liu Y. Zhao L. Wang H. Yang N. Dai S.-S. (2017). Metabotropic glutamate receptor 5 deficiency inhibits neutrophil infiltration after traumatic brain injury in mice. *Sci Rep.* 7:9998. 10.1038/s41598-017-10201-8 PMC5577182 28855570

[B68] Yen T. T. Wong D. T. Bemis K. G. (1987). Reduction of food consumption and body weight of normal and obese mice by chronic treatment with fluoxetine: a serotoninre uptake inhibitor. *Drug Dev. Res.* 10 37–45. 10.1002/ddr.430100106

