# Supplementary material for: Negative Modulation of the Metabotropic Glutamate Receptor Type 5 as a Potential Therapeutic Strategy in Obesity and Binge-Like Eating Behavior
Source: Front Neurosci. 2021 Feb 10;15:631311. doi: 10.3389/fnins.2021.631311 (PMC7902877; doi:10.3389/fnins.2021.631311)
Supplement: Supplementary file 1 [file Table_1.DOC]

**Supplementary material**

**Diets**

RHOSTER® INDÚSTRIA E COMÉRCIO LTDA.

| **Macronutrient** | **Control Diet** | **High-Fat Diet** |
| --- | --- | --- |
| Protein | 20.21 % | 19.0% |
| Carbohydrate | 69.76 % | 36.2% |
| Fat (Total) * | 10.03 % | 44.8% |

Ingredients: Milk casein, Dextrinized starch, Celulose, Soy oil, Saccharose, L-cystine, Animal fat, Mineral mix AIN-93G, Vitamin mix AIN-93, Dibasic calcium phosphate, Corn starch, Choline bitartrate

* Approximate fat profile: 36% saturated fatty acids, 47% monounsaturated fatty acids, 17% polyunsaturated fatty acids

**Induction of binge-like eating by intermittent HFD access**

To assess binge-like eating induced by intermittent HFD access, we used the protocol first published by Czyzyk et al., 2010, and further validated by other published papers [1, 2, 3]. Mice were single housed and randomly assigned to a “continuous HFD/CD access” group, or “intermittent HFD access” group. Information about Rhoster’s HFD and CD are available above.

At the beginning of the protocol, the intermittent group of mice was exposed to both HFD and CD pellets for 48h, and for the next 5 days of the week, only CD pellets were provided. The continuous HFD/CD group had both pellets (HFD and CD) available throughout the experiment. At the beginning of the second, and the following week, the intermittent group had another period of access to both HFD and CD pellets, however for only 24h now. After 24h, HFD was removed and again, only CD was provided for the next 6 days. This cycle of 24h HFD exposure was repeated again before binge-like eating assessment after drugs administration. HFD and CD pellets in the cage were separated by a metal board vertically inserted on the cage’s food holders.

Drugs were administered 30min prior to HFD re-exposure in the intermittent group and at the same time in the continuous HFD/CD group. After 30 min, HFD was made available for continuous and intermittent groups, and food intake was assessed through the difference between initial and final weight of pellets in the cage after 2.5h and 24h.

**DIO mice model**

**Figure S1 – HFD treatment induces an increase on body weight, cholesterol, leptin levels and inflammatory markers.** (A) Representative body weight measurement of mice fed with CD or HFD during 10 weeks. (B) Total serum cholesterol of mice fed with CD or HFD. (C) Serum leptin levels in mice fed with CD or HFD. (D) Epididymal adipose tissue leptin levels of mice fed with CD or HFD (n = 7-8). Graphical representation of cytokines and chemokine concentrations in epididymal adipose of mice fed with either CD or HFD. (E) IL12p70, (F) TNFα, (G) IFNγ, (H) MCP-1, (I) IL-10, (J) IL-6 (n = 6-8). *p<0.05, **p<0.01 and ***p<0.001 compared to control diet (CD).

**Chronic VU0409106 treatment in HFD mice**


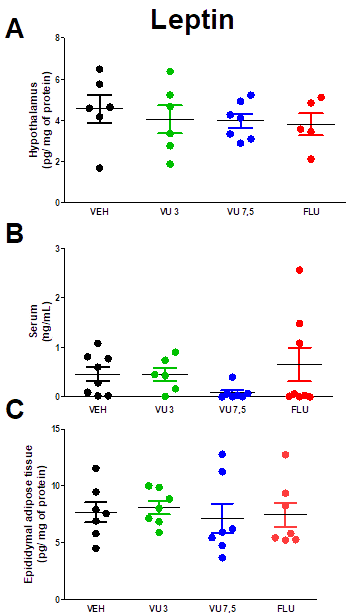


**Figure S2 - VU0409106 had no effect on HFD mice leptin levels.** (A) Hypothalamic leptin, (B) Serum leptin and (C) Epididymal adipose tissue leptin. The results are shown as mean ± SD from n=5-8 individual animals.

**Chronic VU0409106 treatment in HFD mice**

**Figure S3 - VU0409106 had no effect on HFD mice adiponectin levels.** (A) Hypothalamic adiponectin, (B) Serum adiponectin and (C) Epididymal adipose tissue adiponectin. The results are shown as mean ± SD from n=4-9 individual animals.

**Chronic VU0409106 treatment in HFD mice**

**Figure S4 -** **VU0409106 had no effect in HFD mice.** (A) Serum total cholesterol and (B) Serum triglycerides. The results are shown as mean ± SD from n=8-10 individual animals.

**Chronic VU0409106 treatment in HFD mice**

**Figure S5 - VU0409106 had no effect on hypothalamic inflammatory markers levels.** (A) IL12p70, (B) TNFα, (C) IFNγ, (D) MCP-1, (E) IL-10, (F) IL-6. The results are shown as mean ± SD from n=6-8 individual animals.

**Chronic VU0409106 treatment in HFD mice**


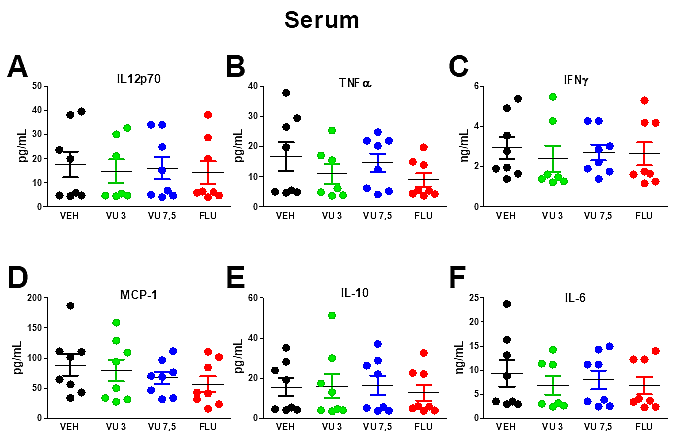


**Figure S6 – VU0409106 had no effect on serum inflammatory markers in HFD mice.** (A) IL12p70, (B)TNFα, (C) IFNγ, (D) MCP-1, (E) IL-10, (F) IL-6. The results are shown as mean ± SD from n=7-8 individual animals.

**Chronic VU0409106 treatment in CD mice**


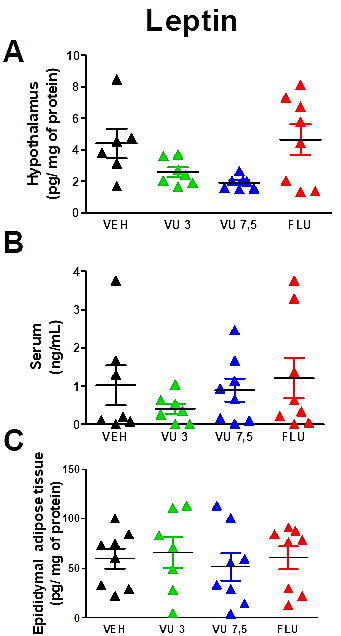


**Figure S7 - VU0409106 had no effect on leptin levels in CD mice.** (A) Hypothalamic leptin, (B) Serum leptin and (C) Epididymal adipose tissue leptin. The results are shown as mean ± SD from n=6-8 individual animals.

**Chronic VU0409106 treatment in CD mice**


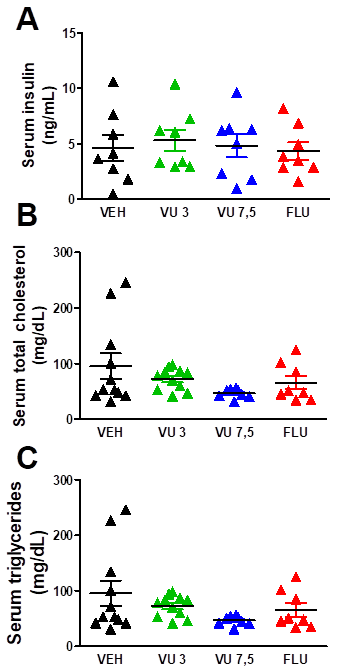


**Figure S8 - VU0409106 had no effect in CD mice.** (A) Serum insulin, (B) Serum total cholesterol and (C) Serum triglycerides. The results are shown as mean ± SD from n=8-11 individual animals.

**Chronic VU0409106 treatment in CD mice**

**Figure S9 - VU0409106 had no effect on adiponectin levels in CD mice.** (A) Hypothalamic adiponectin, (B) Serum adiponectin and (C) Epididymal adipose tissue. The results are shown as mean ± SD from n=3-8 individual animals.

**Chronic VU0409106 treatment in CD mice**

**Figure S10 - VU0409106 reduced IFNγ, but not the other inﬂammatory cytokines in CD mice.** (A) IL12p70, (B)TNFα, (C) IFNγ, (D) MCP-1, (E) IL-6, (F) IL-10. The results are shown as mean ± SD from n=6-8 individual animals.

**Chronic VU0409106 treatment in CD mice**


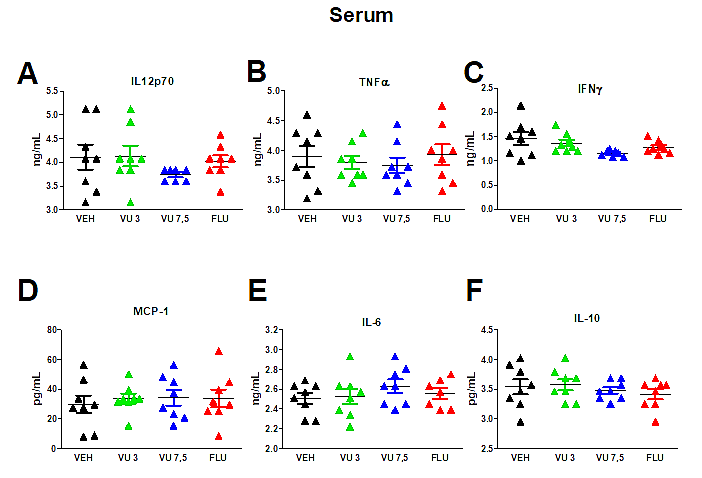


**Figure S11 - VU0409106 had no effect on serum inflammatory markers in CD mice.** (A) IL12p70, (B)TNFα, (C) IFNγ, (D) MCP-1, (E) IL-6, (F) IL-10. The results are shown as mean ± SD from n=7-8 individual animals.

**Chronic VU0409106 treatment in CD mice**

***Figure S12 - VU0409106 had no effect on epididymal adipose tissue inflammatory markers in CD mice.*** (A) IL12p70, (B)TNFα, (C) IFNγ, (D) MCP-1, (E) IL-6, (F) IL-10. The results are shown as mean± SD from n=7-8 individual animals.
